# Protective Effect of *Carnobacterium* spp. against *Listeria monocytogenes* during Host Cell Invasion Using *In vitro* HT29 Model

**DOI:** 10.3389/fcimb.2016.00088

**Published:** 2016-08-26

**Authors:** Tereza Pilchová, Marie-France Pilet, Jean-Michel Cappelier, Jarmila Pazlarová, Odile Tresse

**Affiliations:** ^1^Department of Biochemistry and Microbiology, Faculty of Food and Biochemical Technology, University of Chemistry and TechnologyPrague, Czech Republic; ^2^UMR1014 SECALIM, INRA, OnirisNantes, France

**Keywords:** foodborne pathogens, *Carnobacterium divergens*, *Carnobacterium maltaromaticum*, bacteriocin, HT29, mucus layer

## Abstract

The pathogenesis of listeriosis results mainly from the ability of *Listeria monocytogenes* to attach, invade, replicate and survive within various cell types in mammalian tissues. In this work, the effect of two bacteriocin-producing *Carnobacterium* (*C. divergens* V41 and *C. maltaromaticum* V1) and three non-bacteriocinogenic strains: (*C. divergens* V41C9, *C. divergens* 2763, and *C. maltaromaticum* 2762) was investigated on the reduction of *L. monocytogenes* Scott A plaque-forming during human infection using the HT-29 *in vitro* model. All *Carnobacteria* tested resulted in a reduction in the epithelial cell invasion caused by *L. monocytogenes* Scott A. To understand better the mechanism underlying the level of *L. monocytogenes* infection inhibition by *Carnobacteria*, infection assays from various pretreatments of *Carnobacteria* were assessed. The results revealed the influence of bacteriocin production combined with a passive mechanism of mammalian cell monolayers protection by *Carnobacteria*. These initial results showing a reduction in *L. monocytogenes* virulence on epithelial cells by *Carnobacteria* would be worthwhile analyzing further as a promising probiotic tool for human health.

## Introduction

*Listeria monocytogenes* is one of the major foodborne pathogens responsible for listeriosis. The infections in high-risk individuals, such as pregnant women, newborn infants and immunocompromised people may cause meningitis, encephalitis, septicemia or spontaneous late-term abortions with a mortality rate as high as 30% (Farber and Peterkin, 1991; Rocourt et al., 2000; Vázquez-Boland et al., 2001; Swaminathan and Gerner-Smidt, 2007). In 2013, 1763 human cases of listeriosis were reported in Europe by the European Food Safety Authority and the European Center for Disease Prevention and Control (EFSA and ECDC, 2015). The notification rate was 0.44 cases per 100,000 population which represented an 8.6% increase compared with 2012 (EFSA and ECDC, 2015). There was a statistically significant increase in the incidence of listeriosis over the period 2009–2013. In total, 191 deaths due to listeriosis were reported in 2013, with the highest number (64 cases) occurring in France. *L. monocytogenes* is therefore of public concern in terms of food safety and regulations to control this microorganism. Currently, antibiotics are the most accepted treatment option for listeriosis infections. As vaccination is unavailable and the use of antibiotics is declining due to an increase in resistance and allergies, there is a need for innovative alternative ways of reducing *L. monocytogenes* infections in humans.

*L. monocytogenes* is wide spread in the environment and has been isolated from various sources such as dairy products, fresh vegetables, and meats (Beresford et al., 2001; Guerrieri et al., 2009). Isolations performed in the food processing environment reveal that *L. monocytogenes* can adhere to inert surfaces and grow as biofilms in diverse areas, such as dead ends, crevices and corner cracks (Kim and Frank, 1995; Tresse et al., 2006, 2007; Shi and Zhu, 2009; Pilchová et al., 2014; Guilbaud et al., 2015). Due to the ability of this pathogenic bacterium to grow in foodstuffs at refrigerated temperatures, efficient control methods are required to limit the risk in ready-to-eat food products with a long shelf life. As increasing numbers of consumers prefer foods without chemical preservatives (Cleveland et al., 2001; Devlieghere et al., 2004), there is an opportunity for methods focusing on the use of a protective culture such as lactic acid bacteria (LAB). The protective LAB could produce antimicrobial metabolites such as lactic acid, diacetyl, hydrogen peroxide and bacteriocin or bacteriocin-like compounds (Lindgren and Dodrogosz, 1990).

Some bacteriocin-producing LAB have demonstrated their efficiency in limiting the growth of *L. monocytogenes* (Harris et al., 1989; Rodriguez et al., 1997; Laukova et al., 1999; Ennahar et al., 2000; Sabia et al., 2002; Tyopponen et al., 2003; Todorov et al., 2011; Amado et al., 2012). *Carnobacterium* spp. are among the bacteriocin-producing bacteria recently studied as protective cultures in foodstuffs. The *Carnobacterium* genus belongs to the family *Carnobacteriaceae* within the order of *Lactobacillales* and currently consists of 12 species (Euzéby, 1997). Two of the *Carnobacterium* species, *C. divergens* and *C. maltaromaticum*, are mainly isolated from the environment and foods (Leisner et al., 2007). Both have been shown to exhibit a wide spectrum of activity against *L. monocytogenes* which has been attributed in some cases to the production of bacteriocins (Pilet et al., 1995; Buchanan and Bagi, 1997; Duffes et al., 1999; Nilsson et al., 1999; Schöbitz et al., 1999; Yamazaki et al., 2003; Józefiak et al., 2010). Attempts to apply extracted bacteriocins against *L. monocytogenes* have been limited due to (i) the loss of activity following bacteriocin purification steps, (ii) variations in bacteriocin production depending on *Carnobacterium* spp. and (iii) variations in susceptibility among *L. monocytogenes* strains (Richard et al., 2003; Brillet et al., 2004). Non-bacteriocinogenic strains have also demonstrated inhibitory activity toward *L. monocytogenes* (Nilsson et al., 2005). Some *in vitro* experiments on the antagonism of various LAB against pathogenic bacteria have been reported (Alemka et al., 2010; Garnier et al., 2010; Messaoudi et al., 2012). Although, the active ingredients were mainly attributed to the production of bacteriocins, the inhibitory effect of *Carnobacteria* on *L. monocytogenes* virulence has not yet been explored.

In this study, we examined the *in vitro* potential of protective *Carnobacterium* strains to assess their effect on the virulence of *L. monocytogenes*. Bacteriocin- and non-bacteriocin-producing *Carnobacterium* strains were tested on cell line models using HT29 and its mucin-producing counterpart HT29-MTX.

## Materials and methods

### Bacterial strains

Two clinical strains of *L. monocytogenes* and five strains of *Carnobacterium* spp. were used in this study. *L. monocytogenes* Scott A (serotype 4b) and *L. monocytogenes* LO28 (serotype 1/2c) were previously isolated from listeriosis outbreaks, *C. divergens* V41, and *C. maltaromaticum* V1 (formerly *C. piscicola*) were isolated from salmon and trout intestine and characterized by Pilet et al. (1995), *C. divergens* V41C9 is a *C. divergens* V41 mutant deficient in divercin production (Richard et al., 2003), *C. divergens* NCDO 2763 and *C. maltaromaticum* (formerly *C. piscicola*) NCDO 2762 (type strain) were obtained from the National Collection of Dairy Organisms (Reading, UK). All strains were stored in cryotubes at −80°C in brain heart infusion broth (BHI) for *L. monocytogenes* strains and in Elliker broth for *Carnobacterium* strains supplemented with 20% glycerol as a cryoprotectant.

### Antimicrobial activity determination

The antimicrobial activity of *Carnobacterium* strains was tested on two *L. monocytogenes* strains using the agar diffusion assay (Pilet et al., 1995). Briefly, the *L. monocytogenes* subculture was grown in BHI broth for 8 h at 37°C and culture was incubated overnight at 37°C. A concentration of 3.5 × 10^7^ cfu ml^−1^ was mixed to the BHI agar. *Carnobacterium* strains were subcultured in Elliker broth for 24 h at 20°C. Cultures were then incubated overnight at 20° and 30°C. Similar concentrations of bacteria were obtained after cultivation at 20° and 30°C (2.6 × 10^9^ cfu ml^−1^ and 1.8 × 10^9^ cfu ml^−1^). The cell-free supernatant of each *Carnobacterium* strain was obtained by centrifugation (8200 *g*, 10 min at 4°C) and 10 μl of the filtered supernatant (untreated supernatant and treated culture supernatant adjusted to pH 6.5) was then spotted onto indicator plates of BHI agar (1%) seeded with 10^6^ cfu ml^−1^ of the target strain. After overnight incubation at 37°C, the presence of a translucent halo corresponding to the absence of *L. monocytogenes* growth was observed.

### Cell line cultures

The human adenocarcinoma cell line HT29 and the mucus-secreting HT29 cells selected by adaptation to methotrexate (HT29-MTX) were used. HT29-MTX cells were obtained from Dr Thécla Lesuffleur (INSERM UMR S 938, Paris France) (Lesuffleur et al., 1993). Cells were routinely grown in 25 cm^2^ plastic tissue culture flasks (Nunc, Life Technologies) in 5 ml of culture medium (Dulbecco's modified Eagle's medium; DMEM; Eurobio, Courtaboeuf, France) supplemented with 10% (v/v) fetal calf serum (Eurobio, Courtaboeuf, France), 2 mM L-glutamine (Eurobio, Courtaboeuf, France) and antibiotics—penicillin 100 IU ml^−1^ and streptomycin 100 μg ml^−1^ (Sigma, France). Antibiotics were routinely added to the culture medium except for virulence assays. Cells were maintained in a humidified incubator (at least 90% RH) at 37°C under 5% (v/v) CO_2_ (SANYO Electric Co., Ltd., Osaka, Japan). The medium was changed every other day.

### *L. monocytogenes* plaque-forming assay (PFA)

The ability of *L. monocytogenes* Scott A to form lysis plaques on cell lines HT29 and HT29-MTX was assessed using the PFA as previously described by Roche et al. (2001). Briefly, cell monolayers were grown until they reached 90% confluence in DMEM supplemented with antibiotics and then without antibiotics for another 24 h. The overnight *L. monocytogenes* cultures, grown in BHI broth, were appropriately diluted in DMEM without antibiotics. The 96-wells were inoculated with 2 to 8 log CFU *L. monocytogenes* per well in triplicate and incubated for 2 h at 37°C in a humidified incubator and treated with 100 μg ml^−1^ gentamicin (Sigma, France). After 1.5 h of incubation, cell monolayers were overlaid with an agarose gel containing 0.48% indubiose (Bio-Rad Laboratories, France) in DMEM supplemented with 10 μg ml^−1^ of gentamicin. The number of plaques was counted with a microscope after 48 h of incubation at 37°C in a humidified incubator. Each experiment was repeated three times from independent cultures for each strain and the results were expressed as the number of plaques obtained for 7 or 8 log *L. monocytogenes* loaded per well.

### Ability of *C. divergens* to adhere to epithelial cells HT29

After cultivation, *C. divergens* V41 and V41C9 were harvested by centrifugation, resuspended in DMEM without antibiotics and serum at a concentration of 10^8^ cfu ml^−1^ and loaded on confluent HT29 and HT29-MTX cell line monolayers. After 1 h or 4 h of incubation (37°C, 5% CO_2_), monolayers were washed three times with PBS to remove nonadherent bacteria and lysed with 0.1% Triton X100 for 15 min. The lysate was then diluted and plated on Elliker agar plates to determine the number of adherent bacteria. Each experiment was performed in duplicate.

### PFA in the presence of *carnobacterium*

*Carnobacterium* strains were grown as described above. Cultures were pelleted by centrifugation at 8200 *g* for 10 min and resuspended in DMEM medium without antibiotics. A total of 100 μl of *Carnobacterium* culture in tissue culture medium was used to coat the cells with 10^9^ cfu ml^−1^ for 1 h or 4 h at 37°C. After the initial incubation period, 100 μl of *L. monocytogenes* culture at 10^7^ or 10^8^ cfu ml^−1^ was added on the cell monolayers and incubated for 2 h at 37°C. Then, the medium was replaced with 100 μl of fresh sterile culture medium containing 100 μg ml^−1^ gentamicin and incubated for another 1.5 h at 37°C. Next, the same steps as mentioned above were performed to count the number of lysis plaques. As controls, *L. monocytogenes* and *Carnobacterium* strains were also tested separately on HT29 and HT29-MTX cell lines. Each experiment was repeated three times from independent cultures for each strain.

### Pretreatment of *carnobacterium* before infection assays

Cell monolayers were originally incubated with probiotic cultures at 10^9^ cfu ml^−1^ in antibiotic-free medium. To cover wide range of active compounds that could potentially affect the listerial invasion, six different pretreatments were applied to the *Carnobacterium* culture: (a) 1 ml of an overnight culture of Carnobacteria strains was centrifuged (8200 *g* for 10 min) and the cells obtained in the pellet were resuspended in 1 ml of DMEM (namely—resuspended cells—RS); (b) 1 ml of an overnight culture of *Carnobacterium* was centrifuged (8200 *g* for 10 min) and the cells obtained in the pellet were washed in 1 ml of DMEM, centrifuged once more and subsequently resuspended in 1 ml of DMEM (namely—washed cells—WS); (c) 1 ml of an overnight culture was heated at 100°C for 5 min, centrifuged (8200 *g* for 10 min) and the cells in the pellet resuspended in 1 ml of DMEM (namely—resuspended heated cells—RHC); (d) 1 ml of an overnight culture was heated at 100°C for 5 min, centrifuged (8200 *g* for 10 min) and the cells were washed in 1 ml of DMEM, centrifuged once more time and then resuspended in 1 ml of DMEM (namely—washed heated cells—WHC); (e) overnight cultures were centrifuged (8200 *g* for 10 min) and cell-free supernatant was adjusted to pH 6.5 and then either untreated (namely—non-filtered supernatant—NFS), or filtered (0.2 μm filter; namely—filtered supernatant—FS).

### Statistical analyses

The data were analyzed using Statgraphics Centurion XVI software (StatPoint Inc., Herndon, Virginia, USA). With the confirmation of a normal distribution for each data set, significant differences were determined using two-sided Student's *t*-test comparisons at a 5% significance level.

## Results

### Effect of bacteriocin-producing *C. divergens* V41 on *L. monocytogenes* using *in vitro* virulence models

**Figure 1:**
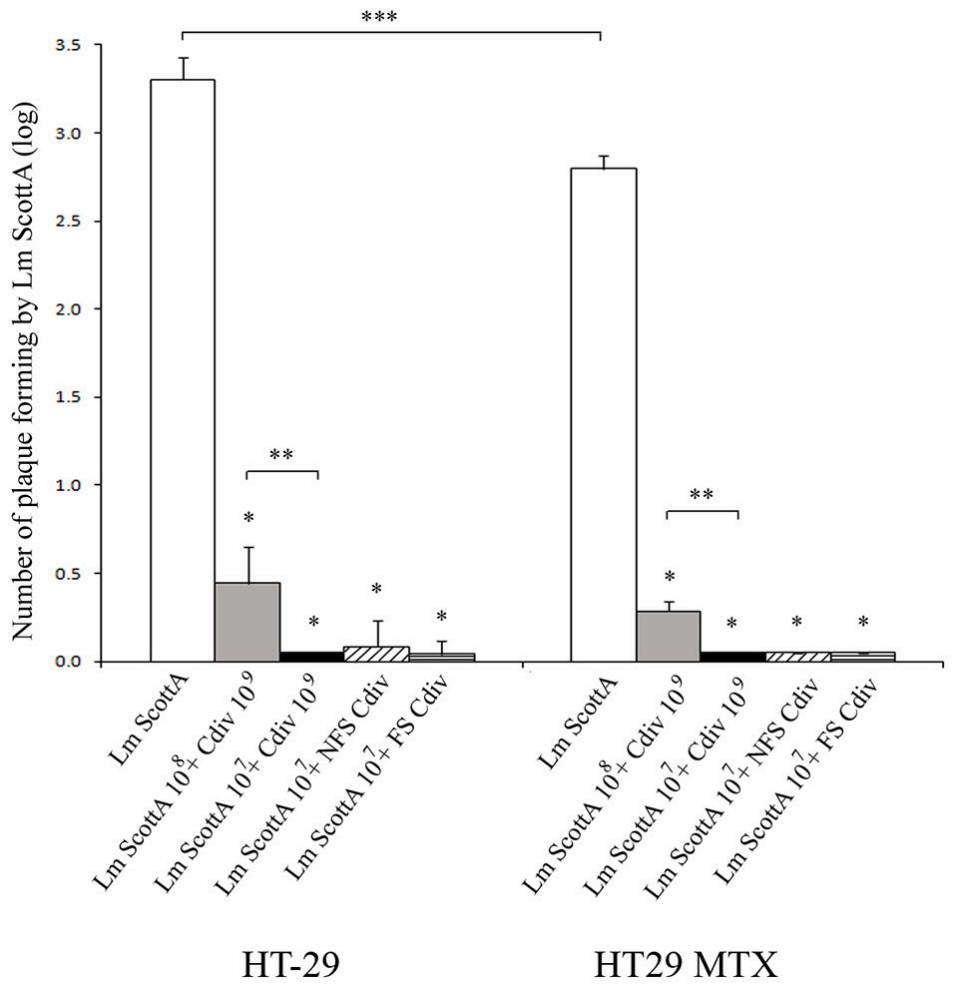
**Inhibition of *L. monocytogenes* Scott A plaque-forming by *C. divergens* V41 using epithelial cells HT29 and mucus-secreting HT29 MTX**. *C. divergens* (Cdiv) was loaded at 10^9^ cfu ml^−1^ 1 h before *L. monocytogenes* Scott A inoculation at 10^7^ or 10^8^ cfu ml^−1^ and incubated for 2 h on HT29 confluent cells. Supernatants (filtered FS or non-filtered NFS) of *C. divergens* culture were also tested. No plaque-forming was observed when Cdiv was loaded alone. Results represent the mean PFA of *L. monocytogenes* Scott A (Lm Scott A) ± SD from at least three independent experiments. Asterisks indicate significant differences (*P* < 0.05) compared to Scott A alone (*), between *L. monocytogenes* inocula (**) and between HT29 and HT29 MTX (***). As no significant difference in plaque-forming was observed after loading Scott A alone at 10^7^ or 10^8^ cfu ml^−1^, these results were pooled (*n* = 6).

First, the antimicrobial activity of the divercin-producing *C. divergens* V41 (div+) and its non-bacteriocinogenic mutant *C. divergens* V41C9 (div−) against *L. monocytogenes* was confirmed (Table 1). As expected, the growth of Scott A and LO28 was inhibited by supernatants of the div+ strain cultivated at 20° and 30°C while no inhibition zone was observed for the div− strain in all conditions. Identical results observed with pH-neutralized supernatants confirmed the divercin action of V41 against *L. monocytogenes* (Table 1). In the following experiments, the effect of *Carnobacteria* against *L. monocytogenes* virulence was assessed using Scott A as it is more virulent than LO28. Plaque-forming on confluent monolayer epithelial cells by *L. monocytogenes* Scott A was evaluated after 1 h of *C. divergens* V41 inoculation (Figure 1). No plaque was observed when *C. divergens* V41 was loaded alone indicating the absence of a cytotoxic effect by *C. divergens* on HT29 and HT29 MTX cell lines. In contrast, *L. monocytogenes* Scott A was able to form plaques on both cell lines with a higher level on HT29 cells (log 3.27 ± 0.12 plaques) than on their mucus-secreting counterparts (log 2.80 ± 0.0.08). The significant difference between the two cell lines indicates a role of mucus in the prevention of *L. monocytogenes* plaque-forming. When Scott A was inoculated on epithelial cells previously coated with *C. divergens* V41, the plaque-forming ability of Scott A was reduced dramatically on both cell lines. At 10^8^ cfu ml^−1^, plaque-forming by Scott A decreased to log 0.44 on HT29 and to log 0.29 on HT29 MTX while at 10^7^ cfu ml^−1^, the plaque-forming of Scott A was reduced to an undetectable level on both lines. Furthermore, when the supernatant (filtered-FS and not filtered-NFS) of V41 culture was used, a similar inhibitory effect on Scott A plaque-forming was obtained on both cell lines.

**Table 1:** **Growth inhibition of *L. monocytogenes* Scott A and LO28 by *C. divergens***.

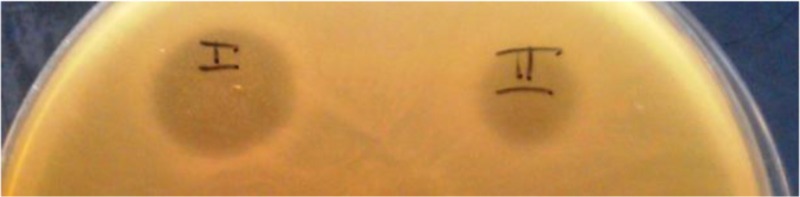		
	**Scott A**	**LO28**
**Untreated supernatant**		
V41 (20°C)	+	+
V41 (30°C)	+	+
V41C9 (20°C)	−	−
V41C9 (30°C)	−	−
**Supernatant adjusted to pH 6.5**		
V41 (20°C)	+	+
V41 (30°C)	+	+
V41C9 (20°C)	−	−
V41C9 (30°C)	−	−

*Supernatants of cultures at 20° and 30°C of the bacteriocin-producing strain C. divergens V41 and its non-bacteriocinogenic variant V41C9 were tested. Growth inhibition was measured using the agar diffusion test as shown in the picture for Scott A with the cell-free culture supernatant of V41 at 20°C (lane I) and at 30°C (lane II). Neutralized supernatants were obtained by adjusting the pH to 6.5*.

*+, inhibition zone ≥ 3 mm; −, inhibition zone < 3 mm*.

### Comparative effect of bacteriocinogenic and non-bacteriocinogenic *C. divergens* strains on *L. monocytogenes* Scott A *in vitro* virulence

In order to determine the contribution of the bacteriocin (divercin) secreted by *C. divergens* V41 to the inhibition of *L. monocytogenes* plaque-forming, the div− mutant, defective in bacteriocin synthesis, was tested. When the HT29 cells were precoated with the div− strain, the inhibitory level of *L. monocytogenes* plaque-forming only decreased to 91.5% indicating a major contribution by another factor besides that of bacteriocin (Table 2). From now on, the antimicrobial effects will be named “the bacteriocin effect” for those due to bacteriocins and “protective effect” for those independent of bacteriocins. The inhibition of plaque-forming by the div− strain was significantly lower than that of div+ on both cell lines confirming a slight but significant contribution of the bacteriocin effect in the div+ strain (Table 2). In addition, a lower plaque-forming inhibition was observed on HT29 MTX for Scott A at 10^8^ cfu/mL which confirms the role of the mucus observed in Figure 1 in preventing *L. monocytogenes* plaque-forming (Table 2).

**Table 2:** **Comparison of the inhibiting efficiency of bacteriocin-producing strain *C. divergens* 41 (div−) and the non-bacteriocin-producing strain V41C9 (div−) on *L. monocytogenes* Scott A plaque-forming on HT29 and mucus-secreting HT29 MTX**.

***C. divergens***	***L. monocytogenes* (cfu ml^−1^)**	**HT29**	**HT29-MTX**
		**Mean ± SD (%)**	**Mean ± SD (%)**
V41 (div+)	Scott A (10^7^)	100.0 ± 0.0	100.0 ± 0.0
V41 (div+)	Scott A (10^8^)	86.5 ± 6.3	89.1 ± 1.7
V41C9 (div−)	Scott A (10^7^)	91.5 ± 6.6^*^	98.7 ± 2.7
V41C9 (div−)	Scott A (10^8^)	74.9 ± 4.4^*^	81.7 ± 3.2^*^

Carnobacteria were inoculated at 10^9^ cfu ml^-1^. HT29 and HT29 MTX epithelial cells were precoated with Carnobacteria 1 h before the inoculation of L. monocytogenes at 10^7^ or 10^8^cfu ml^-1^. Results were normalized to the plaques obtained with L. monocytogenes Scott A alone (log 3.27 ± 0.12, n = 4 on HT29 and log 2.80 ± 0.08, n = 4 on HT29 MTX). The means and standard deviations (SD) were calculated from at least three independent experiments. Asterisks indicate significant differences (P < 0.05) between div+ and div−.

### Influence of precoating time *C. divergens* on the inhibition of *L. monocytogenes* Scott A infection using HT29 and HT29-MTX cell lines

**Figure 2:**
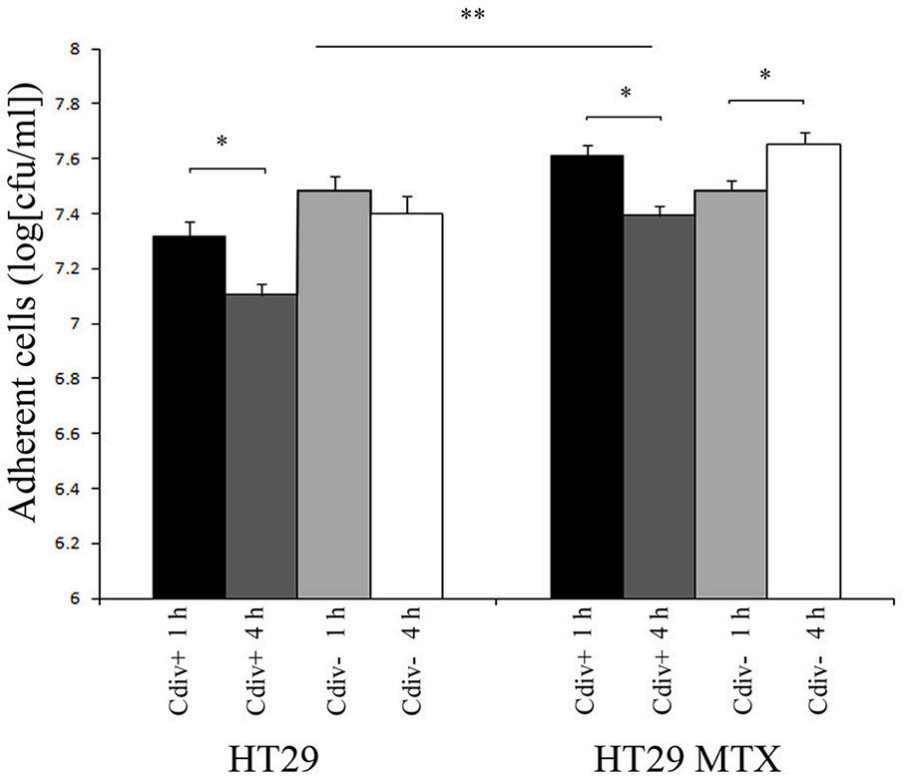
**Viability of *Carnobacterium* strains on epithelial cells after 4 h of contact time with HT29 and HT29-MTX epithelial cell model**. The bacteriocin-producing strain *C. divergens* V41 (div+) and the non-bacteriocinogenic strain V41C9 (div−) were inoculated at 10^9^ cfu ml^−1^. Results represent the mean ± SD from three independent experiments. Asterisks indicate significant differences (*P* < 0.05) between 1 and 4 h of precoating (*) and between HT29 and HT29 MTX (**).

The ability of strains div+ and div− to adhere to host cells was assessed by incubating the culture for 1 h and 4 h on confluent monolayers of HT29 and HT29-MTX (Figure 2). The initial *C. divergens* concentration loaded onto cell monolayers was 10^9^ cfu ml^−1^. Overall, approximately 5 × 10^7^ cfu ml^−1^ of viable *C. divergens* cells were recovered from cell lysates. A significantly greater number of adherent cells was observed on HT29-MTX than on HT29 indicating a positive effect of mucus on *C. divergens* adhesion (Figure 2). In addition, a decrease, although moderate, was observed in the adhesion of div+ after 4 h of contact on both cell lines compared to 1 h while adhesion tended to increase for div− on HT29-MTX. When assessed in the presence of *L. monocytogenes* Scott A, there was no significant difference between 1 and 4 h of contact except for div− after 4 h of contact on HT29 MTX which showed less inhibition of Scott A plaque-forming compared to 1 h (Table 3). Taken together, these results indicate that *C. divergens* V41 remains efficient against Scott A after 4 h of contact with both cell lines by maintaining both its protective and bacteriocin effects.

**Table 3:** **Effect of precoating contact time of *Carnobacterium* strains on the inhibition of *L. monocytogenes* plaque-forming on HT29 and HT29 MTX**.

**Contact time of *C. divergens* (h)**	**HT29**		**HT29 MTX**	
	**1**	**4**	**1**	**4**
V41 (div+)/Scott A	100.0 ± 0.0	97.0 ± 3.1	100.0 ± 0.0	100.0 ± 0.0
V41C9 (div−)/Scott A	91.5 ± 6.6	82.8 ± 9.9	98.7 ± 2.7	82.4 ± 2.6*

L. monocytogenes cells were inoculated at 10^7^ cfu ml^-1^ 1h or 4 h after the inoculation of C. divergens V41 (div+) or V41C9 (div−) at 10^9^ cfu ml^-1^. Results were normalized to the plaques obtained with L. monocytogenes Scott A (log 3.29 ± 0.13, n = 4 on HT29 and log 2.79 ± 0.08, n = 4 on HT29 MTX). The means and standard deviations (SD) were calculated from at least three independent experiments. Asterisks indicate significant difference (P < 0.05) between 1 and 4 h of precoating.

### Effect of various pretreatments of *C. divergens* V41 on the inhibition of *L. monocytogenes* Scott A plaque-forming on HT29

**Figure 3:**
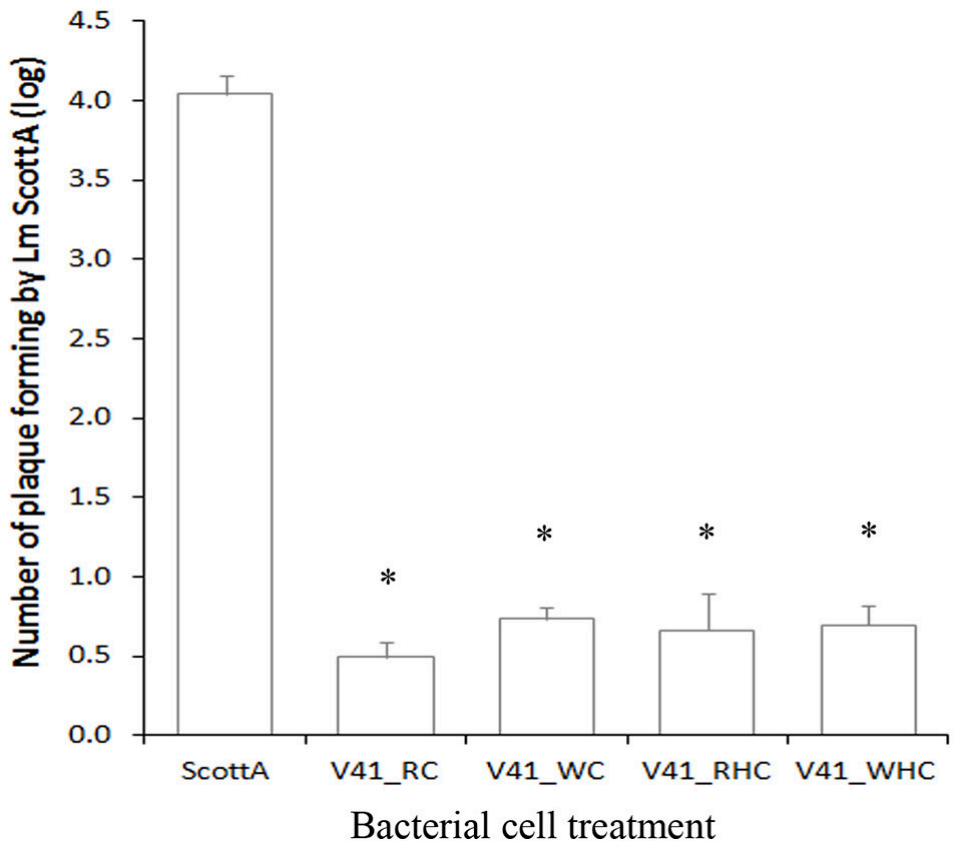
**Inhibition of *L. monocytogenes* Scott A plaque-forming by pretreated *C. divergens* V41 using the HT29 epithelial cell model**. *C. divergens* V41 was inoculated at 10^9^ cfu ml^−1^ 1 h before inoculating *L. monocytogenes* at 10^7^ cfu ml^−1^. (RC) control without pretreatment; (WC) washed cells; (RHC) resuspended heated cells and (WHC) washed heated cells. The results are expressed as mean ± SD from two independent experiments. Asterisks indicate significant differences (*P* < 0.05) compared to Scott A alone. No significant difference was observed between treatments and the control (*P* > 0.05).

To understand further the protective mode of action of *C. divergens* V41, additional infection assays with various pretreatments were carried out (Figure 3). The results showed no significant difference in plaque-forming by *L. monocytogenes* when cells were washed and/or heated before inoculation on HT29, indicating that no extracellular divercin remained after the preparation of the div+ culture and that dead cells were able to achieve the same efficient protective effect.

### Effect of *carnobacterium* spp. on the inhibition of *L. monocytogenes* plaque-forming on HT29

**Figure 4:**
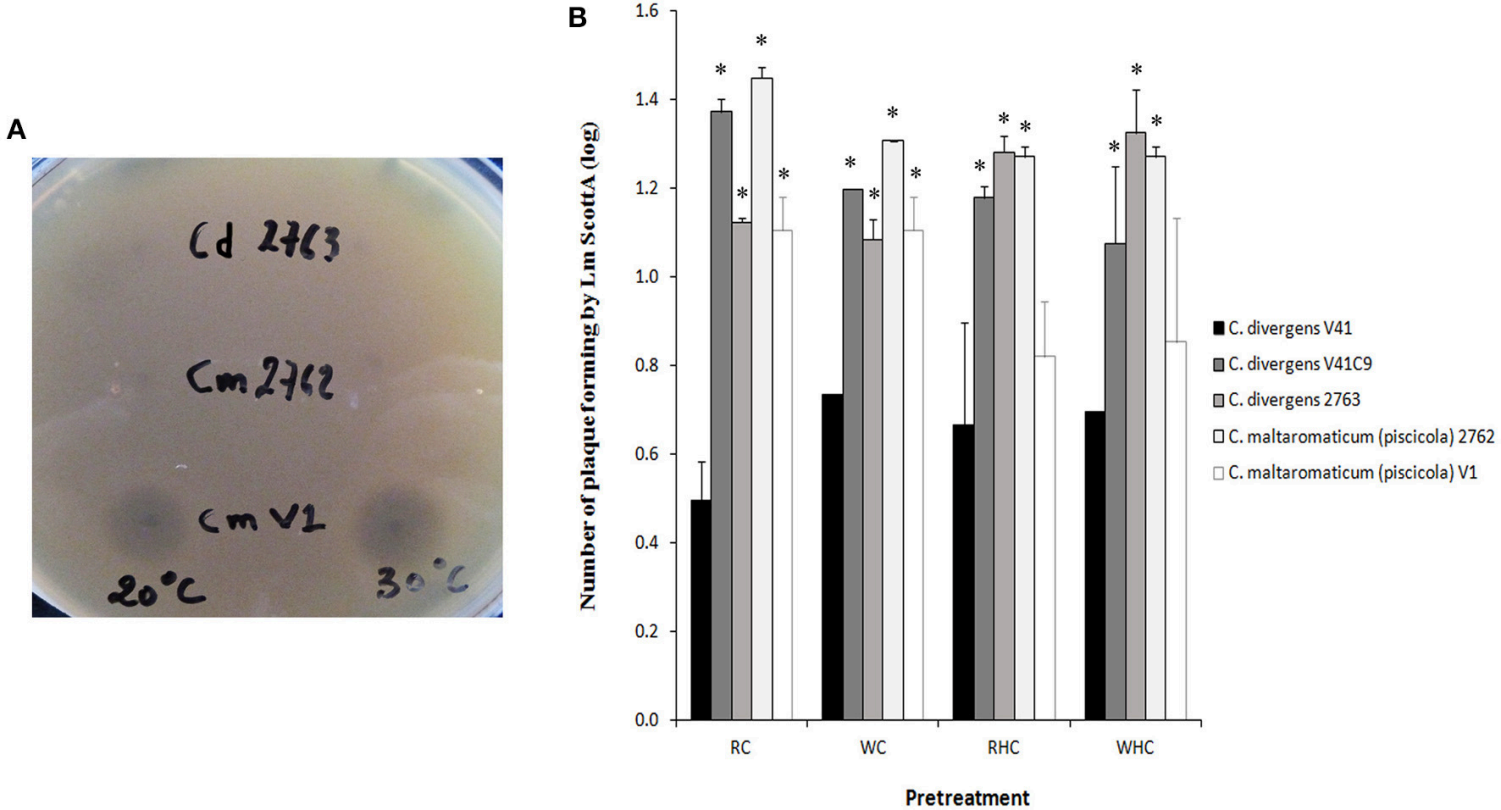
**Inhibition of *L. monocytogenes* Scott A plaque-forming by *Carnobacteria* using the HT29 epithelial cell model**. Two bacteriocin-producing strains (*C. divergens* V41 and *C. maltaromaticum* V1 and three non-bacteriocinogenic strains (*C. divergens* V41C9, *C. divergens* 2763, *C. maltaromaticum* 2762) were compared. **(A)** Capabilities to inhibit growth of *L. monocytogenes* Scott A by *C. divergens* V1, *C. divergens* 2763, *C. maltaromaticum* 2762. Growth inhibition was measured using the agar diffusion test at 20° and at 30°C. Neutralized supernatants were obtained by adjusting pH to 6.5. **(B)** Each *Carnobacterium* strain was inoculated at 10^9^ cfu ml^−1^ on HT29 cells 1 h before loading *L. monocytogenes* at 10^7^ cfu ml^−1^. Pretreatments were as follow: control without pretreatment (RC); washed cells (WC); resuspended heated cells (RHC); and washed heated cells (WHC). The results are expressed as mean ± SD from two independent experiments. Plaque-forming by *L. monocytogenes* Scott A alone was log 3.52 ± 0.21, *n* = 4. All *Carnobacteria* significantly inhibited plaque-forming by *L. monocytogenes* Scott A. Asterisks indicate significant differences (*P* < 0.05) compared to *C. divergens* V41.

In order to determine if the protective effect of *Carnobacterium* against *L. monocytogenes* is specific to *C. divergens* V41, additional strains or species were tested (Figure 4). The anti-listerial activity of cell-free neutralized supernatants of *C. maltaromaticum* V1 was confirmed (inhibition zone = 10 mm ± 0; *n* = 3 at 20°C and 9 mm ± 1; *n* = 3 at 30°C) while the supernatants of non-bacteriocinogenic strains *C. divergens* 2763 and *C. maltaromaticum* 2762 did not exhibit any growth inhibition of *L. monocytogenes* Scott A (inhibition zone < 3 mm; *n* = 3 at 20° and 30°C; Figure 4A). All five *Carnobacterium* strains tested, bacteriocinogenic or not, significantly inhibited the number of plaques formed by *L. monocytogenes* Scott A (Figure 4B). However, the decrease in plaque-forming was lower for all strains compared to that of div+ indicating a better potential of *C. divergens* V41 to prevent *L. monocytogenes* infection. In line with what was observed with V41 and its div− mutant, the bacteriocin-producing *C. maltaromaticum* V1 was significantly more efficient at limiting the number of plaques formed by *L. monocytogenes* than the non-bacteriocin-producing strain *C. maltaromaticum* 2762. Similar results obtained with washed or preheated cells confirmed that inhibition of Scott A plaque-forming by *Carnobacteria* can utilize both bacteriocin and protective effects.

## Discussion

The inhibitory effect of two bacteriocin-producing *Carnobacteria* (*C. divergens* V41 and *C. maltaromaticum* V1) and three non-bacteriocin-producing *Carnobacteria* (*C. divergens* V41C9, *C. divergens* 2763 and *C. maltaromaticum* 2762) against pathogenic *L. monocytogenes* strains has previously been investigated in food (Pilet et al., 1995; Richard et al., 2003; Brillet et al., 2004, 2005). The use of *C. divergens* V41 and *C. maltaromaticum* V1 could represent an alternative strategy to control the growth of *L. monocytogenes* in cold-smoked salmon (Brillet et al., 2004). Overall, LAB isolated from salmon intestine resulted in growth inhibition of *Aeromonas salmonicida* with no reduction in the mortality rate of the fish (Gildberg et al., 1995).

Several methods have been proposed to assess the virulence of *L. monocytogenes*. Mouse infection models have demonstrated their efficiency in differentiating virulent from non-virulent strains (Roche et al., 2001); nonetheless their use is limited due to ethical considerations. Expression of the main virulence genes *hlyA, actA, inlA*, and *prfA* using RT-qPCR has also been investigated to show the effect of environmental conditions on virulence factor transcript levels of *L. monocytogenes* (Duodu et al., 2010). However, the plaque-forming assay using HT29 cells is usually proposed as the best alternative to animal models as it takes into account the epithelial cell invasion capability of the pathogen (Roche et al., 2001). In this work, HT29 cell assays were completed by mucus-secreting HT29 MTX cells in order to include the potential role of mucus in bacterial invasion capability.

A significant reduction in *L. monocytogenes* virulence on epithelial cells was observed when the cell monolayers were precoated with *C. divergens* V41 cultures during 1 or 4 h. The capability to limit foodborne pathogen virulence has previously been tested for probiotic LAB and found to be strain-specific. For instance, Garriga et al. (2015) reported that only bacteriocinogenic *Lactobacillus sakei* of 5 other LAB tested significantly reduced the adhesion of *L. monocytogenes*. *Lactobacillus* and *Bifidobacterium* were also shown to inhibit significantly the subsequent listerial infection using the *in vitro* C2Bbe1 epithelial cell model (Corr et al., 2007). Pretreatment of intestinal cells T 84 with LAB prevents injury of *Escherichia coli* O157:H7 and *E. coli* O157:H6 induced by attaching-effacing-*Lactobacillus* species (Sherman et al., 2005). In the case of *Campylobacter jejuni*, the leading cause of bacterial foodborne diseases (EFSA and ECDC, 2015; Turonova et al., 2015), adhesion, internalization and translocation of HT29 cells were attenuated by strains of *Lactobacillus rhamnosus, Lactobacillus helveticus*, and *Lactobacillus salivarius* (Alemka et al., 2010). The authors reported that live LAB and prolonged precolonization of mammalian cells with probiotics is a prerequisite for probiotic action against *Campylobacter* virulence. More recently, Srividya et al. (2015) demonstrated an *in vitro* inhibition of 70% of *Shigella dysentariae* by a probiotic lactic acid bacterial lysate.

In this study, to investigate the mechanism that could be involved in the efficiency of counteracting *L. monocytogenes* Scott A by *Carnobacterium*, comparisons were made between the divercin-producing *C. divergens* V41 (div+) and *C. divergens* V41C9 (div−), a mutant defective in divercin production (Richard et al., 2003). As expected, div− did not show any inhibitory effect on *L. monocytogenes* cultured on plates in accordance with previous studies (Pilet et al., 1995; Richard et al., 2003). In addition, div+ supernatants resulted in a similar inhibitory effect on plaque-forming by *L. monocytogenes* indicating the efficiency of divercin on *L. monocytogenes* during invasion assays. Nonetheless, both div+ and div− were able to reduce dramatically *L. monocytogenes* plaque-forming on HT29 and HT29 MTX cell lines. The effect of the div+ strain was slightly but significantly higher than that obtained with div− indicating the contribution of bacteriocin activity by the div+ strain since the adhesion rate of both protective cultures was similar on both cell line models. Another bacteriocin-producing *C. maltaromaticum* V1 was also more efficient at limiting the number of plaques formed by *L. monocytogenes* than the non-bacteriocinogenic strain counterpart (*C. maltaromaticum* 2762). This work indicates that non-bacteriocinogenic *Carnobacteria* strains are also effective candidates for limiting the pathogenicity of *L. monocytogenes* using the combined effects of bacteriocin activity and mammalian cell protection. The preheated cell treatment suggests that the protective effect of *Carnobacteria* could be attributed to a passive mechanism. A significant inhibition of *L. monocytogenes* adhesion, invasion and transepithelial translocation was obtained using *Lactobacillus paracasei* but only if this strain was recombined to obtain the expression of *Listeria* adhesion protein (LAP, Lmo1634) in order to interact specifically with the host cell receptor Hsp60 (Koo et al., 2012). In our study, an inhibitory effect of *L. monocytogenes* virulence by *Carnobacterium* was obtained without genetically engineered strains. This could be explained by an increase in epithelial barrier functions due to an interaction with secreted components (Shen et al., 2005). Inhibition mechanisms could also involve specific proteins that accumulate on the cell-surface as described for *Streptococcus pneumoniae* (Guiral et al., 2005). However, further analyses are required to unravel the protective effect of mammalian cells by *Carnobacteria*.

We also observed that the mucus layer enhanced the impact of the protective effect of *Carnobacteria*. This was correlated to a significantly higher number of adherent cells. Similar results were obtained by Alemka et al. (2010) who reported the contribution of the mucus layer to the potential efficacy of probiotic treatment for the attenuation of *C. jejuni* pathogenicity. Mucus constitutes a physical and chemical protective barrier of epithelial cells. Its complex composition includes electrolytes, plasma proteins, lipids, nucleic acids and a large variety of high molecular weight glycoproteins called mucins that contribute to the viscoelasticity of mucus (Johansson et al., 2011). The mucus is a sheltering interspace for bacteria protecting them from shearing motions due to intestinal peristalsis. In addition, commensal bacteria trapped in the mucus are less motile and could be organized into biofilms reinforcing epithelial cell protection (Zoetendal et al., 2002). Weak interactions between mucus and bacterial cell surfaces such as hydrophilic/hydrophobic bonds or cell appendages such as pili could also contribute to maintaining cells in sheltering interspace (Douillard et al., 2013).

*Carnobacteria* did not alter HT29 cells indicating the absence of cytotoxicity. For their potential use as protective cultures in food, the question of human the safety of these LAB arises. *Carnobacteria* are not known as members of the human gastrointestinal microbial community like several other LAB. Previous studies have shown that they do not present an imminence for human illnesses, nor for nosocomial infections in hospitals (Leisner et al., 2007). The genome sequence of the type strain of *C. maltaromaticum* NCDO2762 (ATCC35586) has shown potential virulence factors but none of the specific virulence factors that are present in *L. monocytogenes* strains and it was thus concluded that there are no human safety concerns for this species (Leisner et al., 2012). The whole genome sequence of *C. divergens* V41 is now being annotated and analyzed in our laboratory for further research on its harmlessness. In addition, *C. maltaromaticum* and *C. divergens* are considered microorganisms with technological beneficial use according to Bourdichon et al. (2012). Recently, *C. divergens* was added to the authoritative list of microorganisms with a QPS status (qualified presumption of safety) validated by EFSA for the safety risk assessment of microorganisms intentionally added to food and feed (EFSA, 2014). In the USA, the Food and Drug Administration (FDA) provides a notice inventory of substances that have been approved in terms of safety namely substances generally recognized as safe (GRAS) based the on specific usage and dosage for each substance. This inventory lists viable bacteria including *C. maltaromaticum* (FDA, 2015).

In conclusion, this work demonstrates the potential probiotic effect of *Carnobacterium* strains to attenuate the pathogenesis of *L. monocytogenes* Scott A. The probiotic mechanism results from a bacteriocin effect combined to a protective effect of mammalian cells. Probiotics have positive effects on human health and general well-being. They have been historically associated with cultured milk and dairy products. More recently, they have been analyzed for their potential to inhibit pathogenic and spoilage microorganisms (Klaenhammer, 2000; Gill and Guarner, 2004; Grover et al., 2012).

## Author contributions

OT conceived the work; MP, JC and OT designed the work; TP performed the experimental work; TP and OT analyzed and interpreted the data; TP drafted the manuscript; OT, MP and JC contributed to the final manuscript.

### Conflict of interest statement

The authors declare that the research was conducted in the absence of any commercial or financial relationships that could be construed as a potential conflict of interest.
